# Increase in biodegradation of paclobutrazol in soils

**DOI:** 10.1186/1753-6561-8-S4-P203

**Published:** 2014-10-01

**Authors:** Ednaldo Santos, Fernanda Vaz, Ester Gouvei

**Affiliations:** 1 Universidade Federal de Pernambuco, Recife, PE, Brazil

## Background

Paclobutrazol is a regulator of vegetal growth that remains active in the soil and can affect the growth and the development of subsequent cultures. Experiments in two soils (A and B) with and without addition of inoculum or mineral medium were performed to investigate the biodegradation of paclobutrazol. The inoculum contained a mixed culture of three strains of bacteria isolated by enrichment and characterized as *Pseudomonas*. Flasks were incubated at ambient temperature, during 40 days. Paclobutrazol was quantified by high performance liquid chromatography. Addition of inoculum and mineral medium increased the biodegradation in both soils. The biodegradation ranged between 8 and 95%. Higher biodegradation was obtained with addition of mineral medium, independently of soil utilized.

## Methodology

Soil samples were collected at 0-20 cm depth in two areas (A and B) with irrigated mango plantation, with history of application, located in experimental stations of EMBRAPA (Empresa Brasileira de Pesquisa Agropecuária), in northeastern Brazil. Experiments, at ambient temperature during 40 days, with and without addition of inoculum or mineral medium were realized. The inoculum contained a mixed culture of three strains of bacteria isolated by enrichment and characterized as *Pseudomonas*. Control experiments also were performed. Paclobutrazol was quantified by high performance liquid chromatography using C-18 column, methanol:water (80:20) and 221 nm.

## Results and conclusion

In control experiments were obtained around 14 % (A) and 8 % (B) of degradation after 40 days. This reduction of paclobutrazol concentration was attributed at native microbiota, since the soils had a history of application. After repeated applications of some pesticides, soil microorganisms become adapted to use the compound as carbon or energy source and they can grow on it [1]. In experiments with addition of inoculum, the biodegradation was 38 % and 29 %, in soils A and B, respectively. Vaz et al. [2], found about 43 % biodegradation, after 14 days, when added the same inoculum. Higher biodegradation observed by these authors was due to experiments to be in saturated soils. However, the results presented here were carried out in unsaturated soils. Low moisture content limits microbial growth and metabolism [3]. Furthermore, in experiments with addition of inoculum occurs loss of microbial viability during inoculation due to drastic changes in environmental conditions [4]. In experiments with addition of mineral medium, the biodegradation was around 95 %, independently of soil. In relation to control experiment, 579 % and 1088 % increase in biodegradation were obtained, in soil A and B, respectively. The increase in salt concentration led to a higher solubilization and consequently the improvement the biodegradation.
